# Reproducing Human Arm Strategy and Its Contribution to Balance Recovery Through Model Predictive Control

**DOI:** 10.3389/fnbot.2021.679570

**Published:** 2021-05-17

**Authors:** Keli Shen, Ahmed Chemori, Mitsuhiro Hayashibe

**Affiliations:** ^1^Neuro-Robotics Laboratory, Department of Robotics, Graduate School of Engineering, Tohoku University, Sendai, Japan; ^2^LIRMM, University of Montpellier, CNRS, Montpellier, France

**Keywords:** arm strategy, model predictive control, energy consumption, ankle capacity, synergetic joint coordination, balance recovery, quiet standing

## Abstract

The study of human balance recovery strategies is important for human balance rehabilitation and humanoid robot balance control. To date, many efforts have been made to improve balance during quiet standing and walking motions. Arm usage (arm strategy) has been proposed to control the balance during walking motion in the literature. However, limited research exists on the contributions of the arm strategy for balance recovery during quiet standing along with ankle and hip strategy. Therefore, in this study, we built a simplified model with arms and proposed a controller based on nonlinear model predictive control to achieve human-like balance control. Three arm states of the model, namely, active arms, passive arms, and fixed arms, were considered to discuss the contributions of arm usage to human balance recovery during quiet standing. Furthermore, various indexes such as root mean square deviation of joint angles and recovery energy consumption were verified to reveal the mechanism behind arm strategy employment. In this study, we demonstrate to computationally reproduce human-like balance recovery with and without arm rotation during quiet standing while applying different magnitudes of perturbing forces on the upper body. In addition, the conducted human balance experiments are presented as supplementary information in this paper to demonstrate the concept on a typical example of arm strategy.

## 1. Introduction

Balance control mechanism of human has been researched to enhance balance ability of human and humanoid robots (Winter, 1995). In specific, principal balance recovery strategies, namely, ankle, hip, and stepping strategies have been studied based on human experiments (Nashner, 1985; Horak and Nashner, 1986; Horak et al., 1990) and artificial systems (Kuo and Zajac, 1993; Kuo, 1995; Shen et al., 2020b). These strategies have been considered as efficient means to help preventing falls and analyze the mechanism of balance control during standing and walking motions in human rehabilitation and humanoid robot control. For instance, human upright posture (UP) dynamic stability with a simplified inverted model or hip-ankle model has been studied based on bifurcation analyses to improve balance ability related to fall prevention and rehabilitation (Chagdes et al., 2013; Chumacero et al., 2018, 2019). Additionally, arm strategy has been considered as an efficient means to contribute to balance control and reduce the effects of a fall (Marigold and Patla, 2002; Roos et al., 2008; Pijnappels et al., 2010; Shen et al., 2020a).

Many studies related to the arm strategy have been conducted through human experiments and simulations. Cordo and Nashner (1982) studied rapid postural adjustment associated with a class of voluntary movements, including arm rotation, that disturb the postural balance. Ledebt (2000) concluded that arm postures help stabilize the body to maintain the upright position and that balance control improves because of the arm movement. Furthermore, he considered maximization of the gait efficiency based on an organism's propensity for convergence toward a stable coordination between the arms and legs. Atkeson and Stephens (2007) studied optimal control with boundary constraints from one optimization criterion to realize a multi-link model balance control and observed the movement of shoulder joints for the different perturbations. Aoustin et al. (2008) showed that arm swinging can help minimize the energy consumption during walking. Nakada et al. (2010) reviewed the mechanism of arm strategy for balance recovery and proposed Q-learning to produce appropriate arm control torques for humanoid. They concluded that the arm rotation strategy can widen the range of perturbation impulses. Bruijn et al. (2010) studied the influence of arm swinging on balance control for a perturbation as well as the local and global stability of the steady-state gait and concluded that arm movements contributed to the overall stability of human gait. Milosevic et al. (2011) estimated the effectiveness of arm motions in clinical balance and mobility. Boström et al. (2018) verified that in a dynamic balance task during challenged locomotion, the contribution of the upper body motions, particularly the one of arm movements, to human balance regulation increases with the difficulty of the task. The considered balance recovery tasks are in anteroposterior (A/P) direction. Objero et al. (2019) showed that arm movements are important for the control of mediolateral (M/L) postural sway, based on human experimental data.

It is worth noting that all the previous works did not cover the verification of the arm strategy with multiple cases, e.g., active arms, passive arms, and fixed arms, in their human experiments to discuss the usefulness of arm rotations. To our best knowledge, these arm strategies are relevant for stability improvement and energy efficiency in human and humanoid/bipedal walking and standing. Furthermore, they did not leverage nonlinear model predictive control (NMPC) for addressing multiple constraints of the ankle, hip, arm joint angles, and torques and reproducing human-like balance recovery controller in their artificial systems. The features of NMPC consistent with the capacity of the human body and brain such as constraints handling, predictive horizon, optimization, and robustness are not considered very well in all the previous work.

Therefore, we further developed the mechanism of arm strategy for balance recovery based on previous works and compared the results with human balance recovery experimental results. The contributions of our study are summarized as follows.

1. A three-joint, five-link model is built to represent the human body structure for studying quiet standing balance recovery in the A/P direction. This model includes the foot, the lower body, the upper body, and the arms.
2. An NMPC with the system states and the input constraints is proposed from a neuroscience perspective to reproduce human-like balanced behavior evoked by the human central nervous system.
3. Various indexes are verified to evaluate the capability of balance recovery. The root mean square (RMS) deviation and energy consumption are compared for different cases, namely, active arms, passive arms, and fixed arms. These three cases of arm usages are recruited for balance recovery. The obtained data indicate that balance recovery with active arms is the most effective strategy, and balance control with arm usage is better than that without arm usage.
4. Phase portraits of joint angles and whole body center of mass (WB-CoM) are considered to analyze the control pattern of balance recovery motion.
5. Ankle torque boundary constraints are set with different values. Besides, the relationship between ankle capacity and active arm usage is discussed since in our daily life ankle is easy to be injured, we want to observe how arm usages contribute to balance in this case.
6. By comparing the results of the numerical simulation and human experiments, human-like balance recovery with arm strategy is implemented and arm movements are found to enhance the capability of balance recovery.

The paper is organized as follows. In section 2, the simplified model with three different arm usages and their dynamic equation are introduced first. And, the balance recovery controller based on NMPC is proposed in section 2.2. In section 3, the results of simulation and human experiments are discussed to verify if actuated arm usage contributes to balance control. The conclusions of this study are summarized as well as future work in section 4.

## 2. Models and Methods

### 2.1. Dynamic Equation of Simplified Models

**Figure 1:**
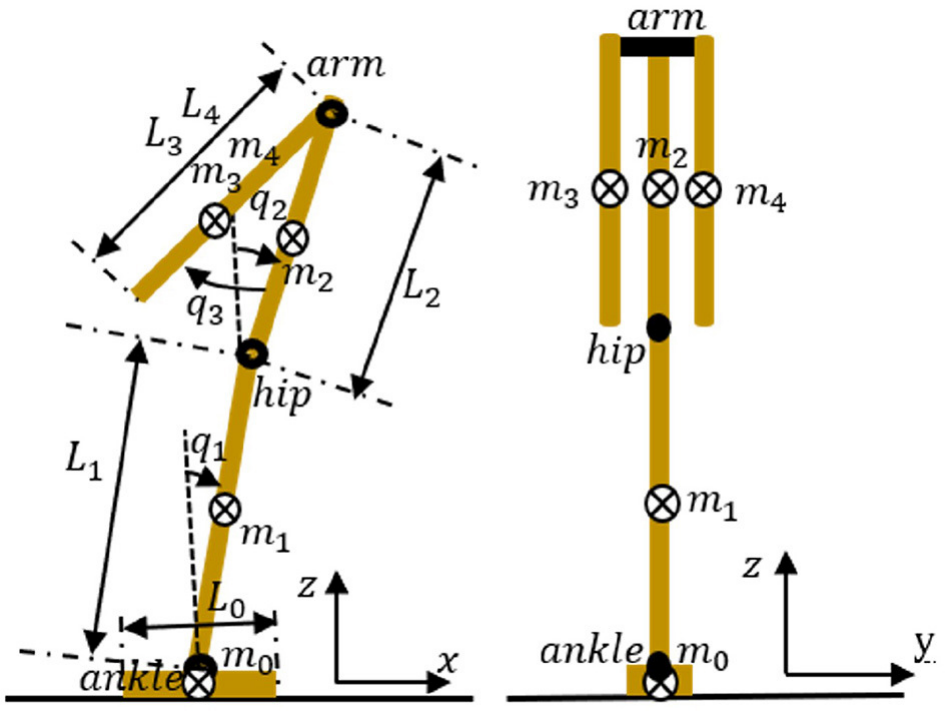
Structure of the three-joint and five-link model. *m*_4_, *m*_3_, *m*_2_, *m*_1_, and *m*_0_ represent the masses of the left arm, right arm, upper body, lower body, and foot, respectively. *L*_4_, *L*_3_, *L*_2_, *L*_1_, and *L*_0_ represent the lengths of the left arm, right arm, upper body, lower body, and foot, respectively. *q*_3_, *q*_2_, and *q*_1_ represent the left-right arm angle, hip angle, and ankle angle, respectively. Here, the right arm and left arm share the same joint motor.

To achieve quiet standing balance control, we regard the human body structure as a simplified three-joint and five-link model consisting of left-right arm joint, hip joint, ankle joint, and right arm, left arm, upper body, lower body, fixed foot (e.g., Figure 1). Table 1 summarizes the physical parameters of our model. Based on an existing anthropometric database (Kouchi et al., 2000) and the previous work (Atkeson and Stephens, 2007) dealing with optimization-based balance recovery strategy, the height and mass of the whole-body are 1.7 [*m*] and 69.3 [*kg*], respectively. Further, *m*_4_, *m*_3_, *m*_2_, *m*_1_, and *m*_0_ represent the masses of the left arm, right arm, upper body, lower body, and foot, respectively; *L*_4_, *L*_3_, *L*_2_, *L*_1_, and *L*_0_ represent the lengths of left arm, right arm, upper body, lower body, and foot, respectively; and *q*_3_, *q*_2_, and *q*_1_ represent the left-right arm angle, hip angle, and ankle angle, respectively. Note that the body segments between the head and the left-right arm joint, between the left-right arm joint and the hip joint, and between the hip joint and the ankle joint are ignored.

**Table 1:** Physical parameters of the three-joint, five-link model.

**Link**	**Mass [kg]**	**Length [m]**	**Height [m]**
Foot	1.3	0.3	0.1
Lower body	35	1.0	–
Upper body	25	0.6	–
Right arm	4	0.6	–
Left arm	4	0.6	–
Total mass [kg]	69.3	–	–
Total height [m]	–	–	1.7

First, the dynamic equations of motion for this three-joint, five-link model controlled by the arm, hip, and ankle joint torques are computed based on Lagrange mechanics (Paul, 1981). The Lagrange equations and dynamic equation of motions are derived for the model with three different arm states separately: active arms, passive arms, and fixed arms, as shown in Table 2. In that table, *T* and *V* represent the kinetic and potential energy, respectively; τ_*arm*_, τ_*hip*_, and τ_*ankle*_ represents the arm torque, hip torque, and ankle torque, respectively; *M*_11_, *M*_12_, *M*_13_, *M*_21_, *M*_22_, *M*_23_, *M*_31_, *M*_32_, and *M*_33_ are the inertia terms; and *C*_1_, *C*_2_, and *C*_3_ denote the total centrifugal, Coriolis, and gravity forces.

**Table 2:** Lagrange equations and dynamic equation of motions for the model with three different cases: (1) active arms; (2) passive arms; (3) fixed arms.

**Case**	**Lagrange equations**	**Dynamic equation of motion**
(1)	$\frac{d}{dt}\left(\frac{\partial L}{\partial {\dot{q}}_{1}}\right)-\frac{\partial L}{\partial q_{1}}=\tau_{ankle}$,	$\left[\begin{array}{ccc} M_{11} & M_{12} & M_{13}\\ M_{21} & M_{22} & M_{23}\\ M_{31} & M_{32} & M_{33} \end{array}\right]\left[\begin{array}{c} {\ddot{q}}_{1}\\ {\ddot{q}}_{2}\\ {\ddot{q}}_{3} \end{array}\right]+\left[\begin{array}{c} C_{1}\\ C_{2}\\ C_{3} \end{array}\right]=\left[\begin{array}{c} \tau_{ankle}\\ \tau_{hip}\\ \tau_{arm} \end{array}\right]$
	$\frac{d}{dt}\left(\frac{\partial L}{\partial {\dot{q}}_{2}}\right)-\frac{\partial L}{\partial q_{2}}=\tau_{hip}$,	
	$\frac{d}{dt}\left(\frac{\partial L}{\partial {\dot{q}}_{3}}\right)-\frac{\partial L}{\partial q_{3}}=\tau_{arm}$,	
	*L=T-V*.	
(2)	$\frac{d}{dt}\left(\frac{\partial L}{\partial {\dot{q}}_{1}}\right)-\frac{\partial L}{\partial q_{1}}=\tau_{ankle}$,	$\left[\begin{array}{ccc} M_{11} & M_{12} & M_{13}\\ M_{21} & M_{22} & M_{23}\\ M_{31} & M_{32} & M_{33} \end{array}\right]\left[\begin{array}{c} {\ddot{q}}_{1}\\ {\ddot{q}}_{2}\\ {\ddot{q}}_{3} \end{array}\right]+\left[\begin{array}{c} C_{1}\\ C_{2}\\ C_{3} \end{array}\right]=\left[\begin{array}{c} \tau_{ankle}\\ \tau_{hip}\\ 0 \end{array}\right]$
	$\frac{d}{dt}\left(\frac{\partial L}{\partial {\dot{q}}_{2}}\right)-\frac{\partial L}{\partial q_{2}}=\tau_{hip}$,	
	$\frac{d}{dt}\left(\frac{\partial L}{\partial {\dot{q}}_{3}}\right)-\frac{\partial L}{\partial q_{3}}=0$,	
	*L=T-V*.	
(3)	$\frac{d}{dt}\left(\frac{\partial L}{\partial {\dot{q}}_{1}}\right)-\frac{\partial L}{\partial q_{1}}=\tau_{ankle}$,	$\left[\begin{array}{cc} M_{11} & M_{12}\\ M_{21} & M_{22} \end{array}\right]\left[\begin{array}{c} {\ddot{q}}_{1}\\ {\ddot{q}}_{2} \end{array}\right]+\left[\begin{array}{c} C_{1}\\ C_{2} \end{array}\right]=\left[\begin{array}{c} \tau_{ankle}\\ \tau_{hip} \end{array}\right]$
	$\frac{d}{dt}\left(\frac{\partial L}{\partial {\dot{q}}_{2}}\right)-\frac{\partial L}{\partial q_{2}}=\tau_{hip}$,	
	*L* = *T*−*V*.	

### 2.2. Proposed NMPC for Balance Recovery

**Figure 2:**
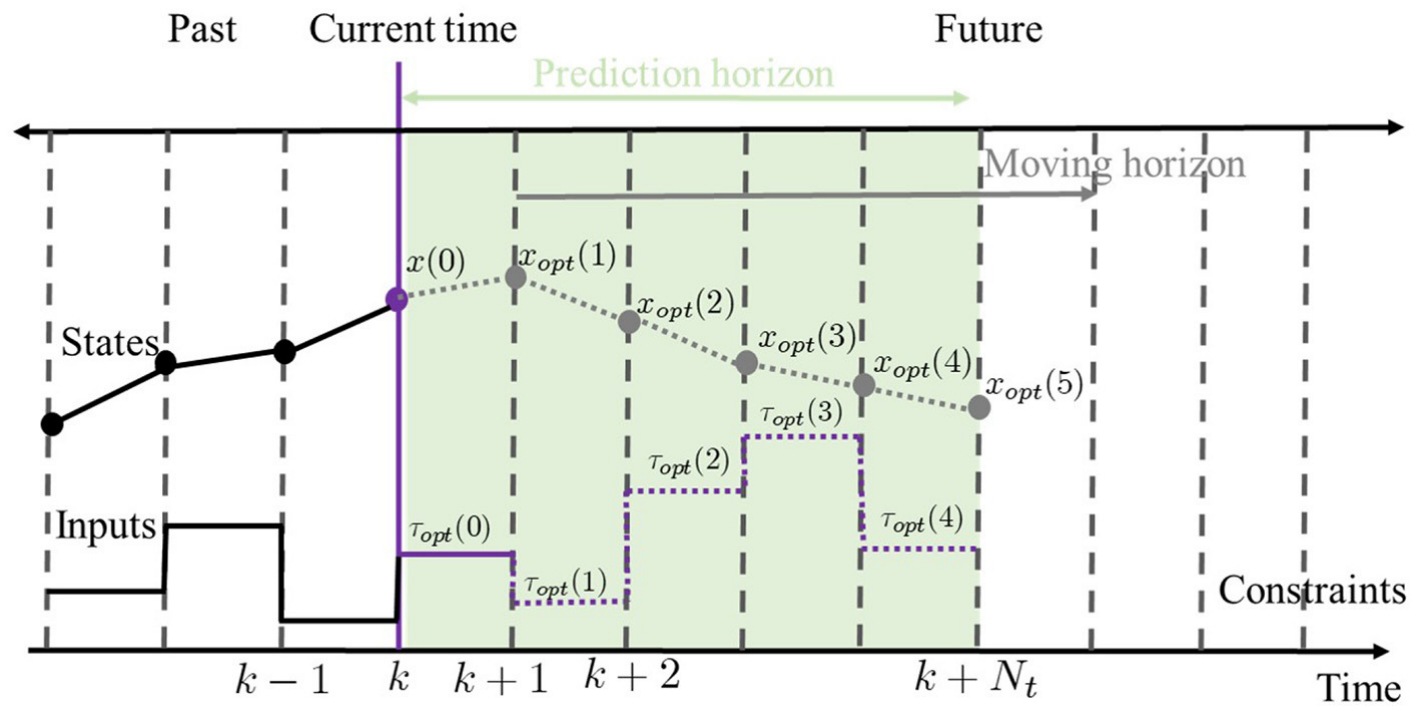
Schematic description of the NMPC at time *k*. The proposed dynamic model is recruited to predict the future motion state sequence ***x***_*opt*_ of the model system and compute optimal control input sequence **τ**_*opt*_ of balance recovery based on the current state through solving an optimization problem. For instance, let NMPC starts at *k* = 0 with a prediction horizon *N*_*t*_ (here *N*_*t*_ = 5) and the initial states ***x***(0).

In this section, an NMPC scheme (Grüne and Pannek, 2017) is proposed to resolve the balance recovery problem. This problem can be solved as an iterative open-loop optimal control problem with a finite horizon and an observable initial states for each sampling time. The procedure of NMPC with constraints is illustrated in Figure 2 to strengthen the NMPC concept explanation. For example, let NMPC starts at *k* = 0 with a prediction horizon *N*_*t*_ (here *N*_*t*_ = 5) and the initial states ***x***(0) = ***x***. The predictive optimal control sequence for the entire horizon can be obtained as follows,

(1) $$\begin{array}{l} \tau_{opt}=\left[\tau_{opt}\left(0\right),\tau_{opt}\left(1\right),\tau_{opt}\left(2\right)\ldots \tau_{opt}\left(N_{t}-1\right)\right] \end{array}$$

The sequence of the predicted states is denoted by,

(2) $$\begin{array}{l} x_{opt}=\left[x_{opt}\left(1\right),x_{opt}\left(2\right)\ldots x_{opt}\left(N_{t}\right)\right] \end{array}$$

Then, the first sample of the optimal control sequence **τ**_*opt*_(0) is applied to the system to produce the state ***x***(1). And, the initial state is updated by ***x***(1) for the new optimal control problem at the sampling time *k* = 1. Then, the above-described optimization process is repeated with the concept of receding horizon (moving horizon) to obtain a new optimal control sequence for the current system. Subsequently, the new initial states can be computed for the next optimal process. Therefore, NMPC is considered as a receding horizon iterative optimal control algorithm.

The cost function considered in the optimal control problem of the NMPC is given by

(3) $$\begin{array}{l} J\left(x\left(0\right),\tau_{\left(0,N_{t}-1\right)}\right)=\overset{N_{t}-1}{\underset{0}{\sum }}l\left(x,k,\tau \right)+V_{f}, \end{array}$$

(4) $$\begin{array}{l} l\left(x,k,\tau \right)=\frac{1}{2}\left(x^{T}\left(k\right)Qx\left(k\right)+\tau^{T}\left(k\right)R\tau \left(k\right)\right), \end{array}$$

(5) $$\begin{array}{l} V_{f}=\frac{1}{2}x^{T}\left(N_{t}\right)Q_{f}x\left(N_{t}\right). \end{array}$$

The penalty weighting dimension and constraints of the NMPC differ for the model with the three different arm states, including active arms, passive arms, and fixed arms.

The cost function (3) is considered such that ***Q***, ***Q***_*f*_, and ***R*** are positive definite symmetric matrices. The states and the control torques can be penalized by tuning ***Q*** and ***R***, respectively. Increasing ***Q*** is aimed to minimize the state tracking error while increasing ***R*** means a reduction of energy consumption. In this research, the ratio between ***Q*** and ***R*** for three cases of arm usages is set as the same value 10^3^ named one optimization criterion (Atkeson and Stephens, 2007). Further, terminal weighting ***Q***_*f*_ = 10^5^ can be used as a tuning parameter to penalize the terminal states to achieve stable NMPC performance.

The objective is to minimize the cost *J*[***x***(0), **τ**_(0,*N*_*t*_−1)_] subject to the following control input and state boundary for the model with three different arm strategies:

(1) NMPC for Model with Active Arms

For *i*_*a*_ = 1, 2, 3, which represent ankle, hip, and arm joints respectively, and *k*_*a*_ = 0, …, *N*_*t*_ − 1, boundary settings of the control inputs have been selected based on the work of Atkeson and Stephens (2007) where a constrained-based optimization is proposed for a multi-balance recovery strategy:

$$\begin{array}{l} \tau_{min}\left(i_{a}\right)\leq \tau_{i_{a}}\left(k_{a}\right)\leq \tau_{max}\left(i_{a}\right), \end{array}$$

where τ_*min*_(1) = −120 [*Nm*], τ_*min*_(2) = −500 [*Nm*], τ_*min*_(3) = −200 [*Nm*], τ_*max*_(1) = 120 [*Nm*], τ_*max*_(2) = 500 [*Nm*], and τ_*max*_(3) = 200 [*Nm*].

For all *i*_*a*_ = 1, …, 6 and *k*_*a*_ = 0, …, *N*_*t*_, the states including angles and angular velocities of ankle, hip, and arm joints are bounded by

$$\begin{array}{l} x_{min}\left(i_{a}\right)\leq x_{i_{a}}\left(k_{a}\right)\leq x_{max}\left(i_{a}\right), \end{array}$$

where *x*_*min*_(1) = −0.2 [*rad*], *x*_*min*_(2) = −0.35 [*rad*], *x*_*min*_(3) = −2.5 [*rad*], *x*_*min*_(4) = −∞ [*rad*/*s*], *x*_*min*_(5) = −∞ [*rad*/*s*], *x*_*min*_(6) = −∞ [*rad*/*s*], *x*_*max*_(1) = 0.4 [*rad*], *x*_*max*_(2) = 1.3 [*rad*], *x*_*max*_(3) = 0.5 [*rad*], *x*_*max*_(4) = ∞ [*rad*/*s*], and *x*_*max*_(5) = ∞ [*rad*/*s*], *x*_*max*_(6) = ∞ [*rad*/*s*]. It is necessary to point out that the three first elements of *x* denote joint angles, and the three last elements represent angular velocities; this is why the unit changes from [*rad*] to [*rad*/*s*]. We just put negative infinity in boundary settings for implementation purposes to keep a wide range of velocity values. However, based on the obtained results, the evolution of the velocities remains very reasonable, i.e., within the interval [−1.2, 1.2] as it can be observed from **Figures 9–11**.

(2) NMPC for Model with Passive Arms

For all *i*_*p*_ = 1, 2 representing the notation of ankle and hip joints, respectively and *k*_*p*_ = 0, …, *N*_*t*_ − 1, the control inputs are bounded by

$$\begin{array}{l} \tau_{min}\left(i_{p}\right)\leq \tau_{i_{p}}\left(k_{p}\right)\leq \tau_{max}\left(i_{p}\right), \end{array}$$

where τ_*min*_(1) = −120 [*Nm*], τ_*min*_(2) = −500 [*Nm*], τ_*max*_(1) = 120 [*Nm*], and τ_*max*_(2) = 500 [*Nm*].

For all *i*_*p*_ = 1, …, 6 representing joint angles and angular velocities of ankle and hip, arm, and prediction horizon *k*_*p*_ = 0, …, *N*_*t*_, the states are bounded by the same constraint settings as the case with active arms.

(3) NMPC for Model with Fixed Arms

For all *i*_*f*_ = 1, 2 representing ankle and hip joints respectively and *k*_*f*_ = 0, …, *N*_*t*_ − 1, the control inputs are bounded by the same constraint settings as the case with passive arm.

For all *i*_*f*_ = 1, …, 4 representing joint angles and angular velocities of ankle and hip, and prediction horizon *k*_*f*_ = 0, …, *N*_*t*_, the system states are bounded by

$$\begin{array}{l} x_{min}\left(i_{f}\right)\leq x_{i_{f}}\left(k_{f}\right)\leq x_{max}\left(i_{f}\right), \end{array}$$

where *x*_*min*_(1) = −0.2 [*rad*], *x*_*min*_(2) = −0.35 [*rad*], *x*_*min*_(3) = −∞ [*rad*/*s*], *x*_*min*_(4) = −∞ [*rad*/*s*], *x*_*max*_(1) = 0.4 [*rad*], *x*_*max*_(2) = 1.3 [*rad*], *x*_*max*_(3) = ∞ [*rad*/*s*], and *x*_*max*_(4) = ∞ [*rad*/*s*].

With the system states and the input constraints, an NMPC is proposed from a neuroscience perspective to reproduce human-like balanced behavior evoked by the human central nervous system. The proposed NMPC also has a predictive aspect that allows predicting the future behavior and computes an optimal control balance strategy by minimizing systemic energy consumption of the whole body. Furthermore, the NMPC technique can handle simultaneously the state and input constraints, which is important to meet realistic requirements due to physical limitations of the human body such as joint ranges and torques saturation. All the previously proposed control techniques can not take into account constraints naturally. In this research, we proposed NMPC which can naturally take into account constraints. Different magnitudes of disturbing forces are applied to the model to observe the autonomous switch between the ankle, hip, and arm strategies and to examine the robustness of the proposed solution.

## 3. Results of Simulation and Discussion Compared to Human Experiments

### 3.1. Simulation Parameter Setting

**Figure 3:**
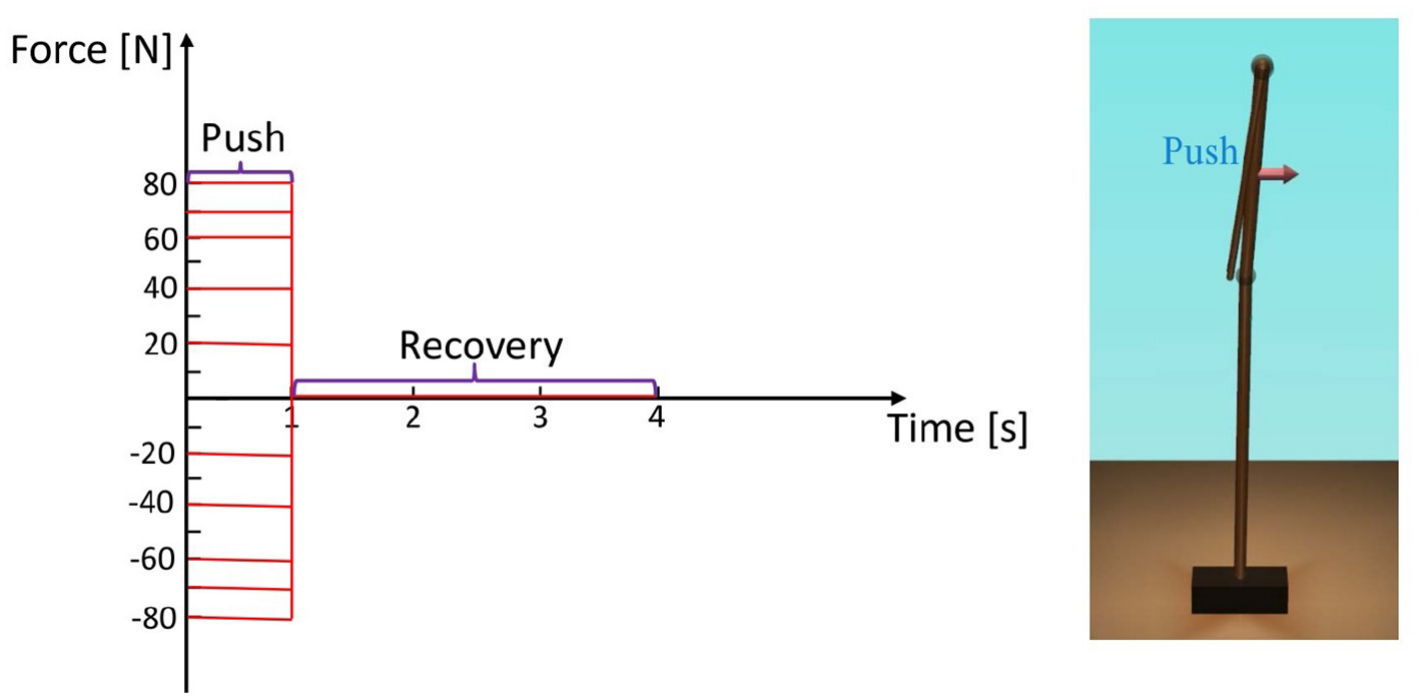
The common simulation settings for the three different arm states.

In this section, we analyze the model motion intensity using the total RMS deviation of the joint angles to verify the effectiveness of the arm strategy. The simulation settings are demonstrated in Figure 3. We pushed the position of the center of mass of the upper body with different disturbing forces backward and forward for 1 [*s*], which could be different with the previous study on perturbation setting with a balance board (Chumacero and Yang, 2019, 2020). The maximum simulation time is set as 4 [*s*] that can make the model finish the process of balance recovery. The disturbing forces were as follows (Atkeson and Stephens, 2007):

1. Push backward: −20 [*N*], −40 [*N*], −60 [*N*], −70 [*N*], and −80 [*N*].
2. No force: 0 [*N*].
3. Push forward: 20 [*N*], 40 [*N*], 60 [*N*], 70 [*N*], and 80 [*N*].

### 3.2. Simulation Results and Discussion

NMPC controller produces predictive ankle-hip strategy after a perturbation while arm strategy can be employed only for the model of active arm setting. For the disturbing forces −80 [*N*] and 80[*N*], only the model with the active arm can realize balance recovery from the unstable states. The models with passive arm and fixed arm are unable to obtain a solution for balance control under the same disturbing force. This indicates that the active arm rotation strategy widens the range of the disturbing forces; this result is similar to the conclusions derived in Nakada et al. (2010) and Kuindersma et al. (2011).

**Figure 4:**
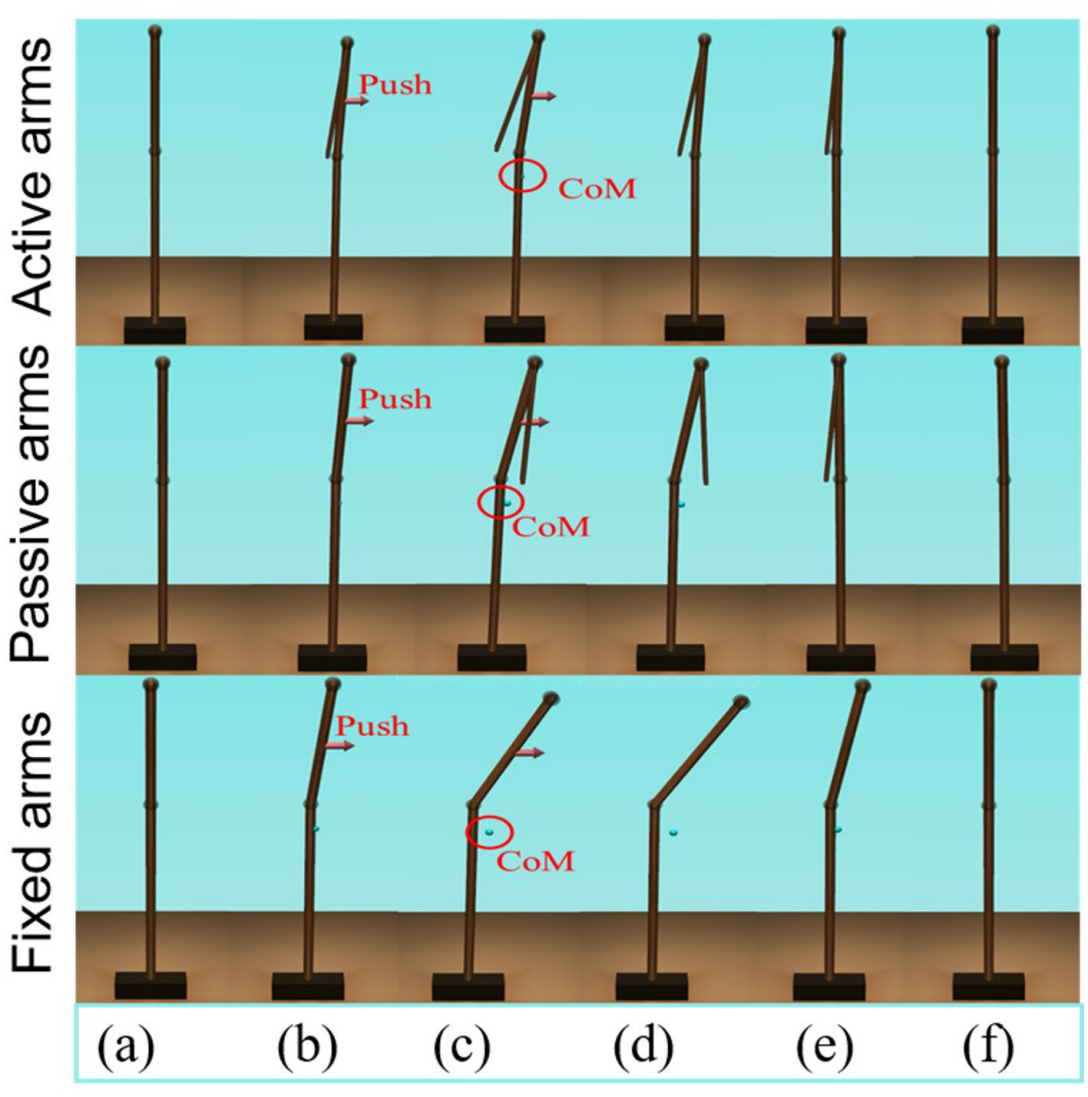
Schematic of the balance behavior for the three different arm states (“top to bottom: active arms, passive arms, and fixed arms”), for a disturbing force of 70 [*N*]. **(A,F)** Represent the equilibrium states; **(B)** represents the pushing forward process; **(C–E)** represents the balance recovery behavior.

**Figure 5:**
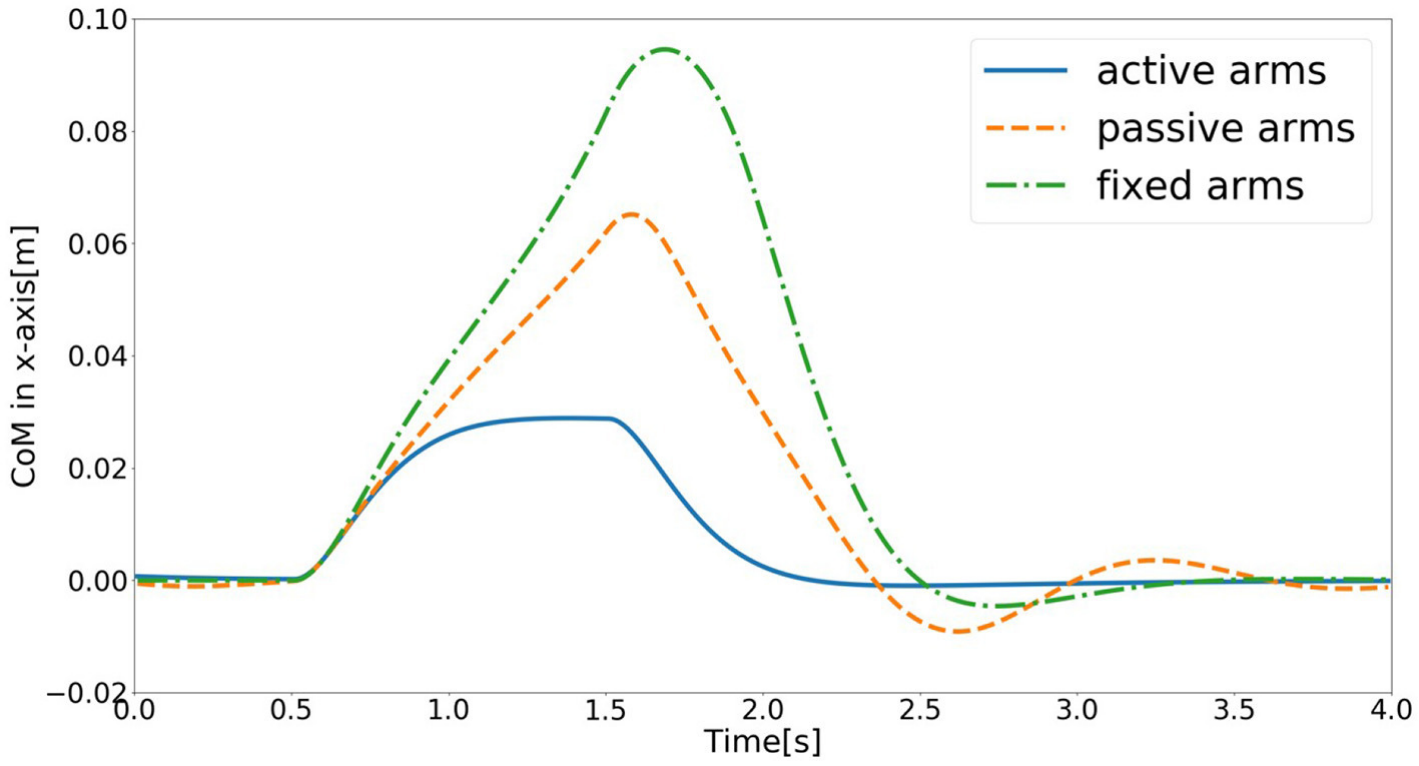
Evolution of center of mass of the model in the x-axis direction for the three different arm states (active arms, passive arms, and fixed arms) for a disturbing force of 70 [*N*].

**Figure 6:**
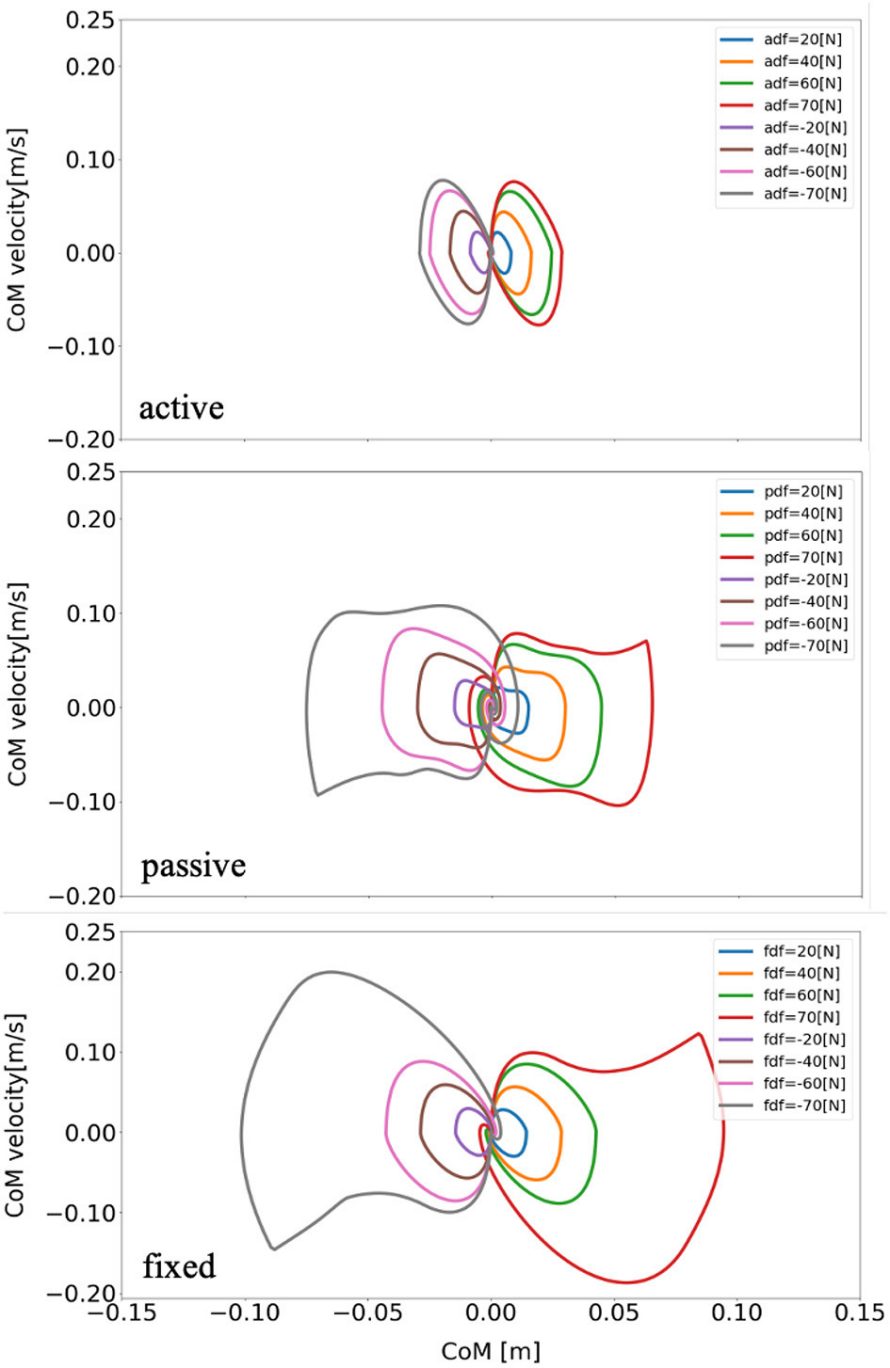
Evolution of the center of mass phase portrait of the model for three different arm states (active arm, passive arm, fixed arm) for different disturbing forces: (1) Push backward: −20 [*N*], −40 [*N*], −60 [*N*], and −70 [*N*]. (2) No force: 0 [*N*]. (3) Push forward: 20 [*N*], 40 [*N*], 60 [*N*], and 70 [*N*]. “a,” “p,” and “f” in the labels “adf,” “pdf,” and “fdf” represent the cases with active arms, passive arms, fixed arms, respectively, and “df” represents the disturbing forces.

The schematic of the movements of the models with active, passive, and fixed arms for a disturbing force of 70 [*N*] is illustrated in Figure 4. The figure shows that the model with active arms has a better ability to realize balance recovery than the other two models. This is because the deviation of the center of mass of the model with active arm usage in the x-axis direction (e.g., Figure 5) is less than the other with passive and fixed arm usages. Figure 6 shows the center of mass for three different arm states is located within the stable region according to the evolution of the whole body center of mass (CoM) velocity vs. its position. Based on the obtained results from this Figure, we concluded that it is located within the stable region. It is worth noting that the size of CoM phase portraits for the model with active arm usage is smaller than those for the model with passive or fixed arm usage. This indicates that active arm usage can maintain the center of mass of the body to remain close to the origin (equilibrium point). From the stability aspect, active arm usage shows more advantage in balance recovery tasks by comparing the deviation of the center of mass.

The total RMS deviation can be calculated by

$$\begin{array}{l} Total\;RMS\;deviation=\sqrt{\frac{1}{N}\overset{N}{\underset{t=1}{\sum }}\left(q_{1}{\left(t\right)}^{2}+q_{2}{\left(t\right)}^{2}\right)}\;, \end{array}$$

where, *N* denotes the total samples number, which can be computed from the recovery time and the sampling period, *q*_1_(*t*) and *q*_2_(*t*) represent the ankle and hip angles at each sampling point, respectively.

**Figure 7:**
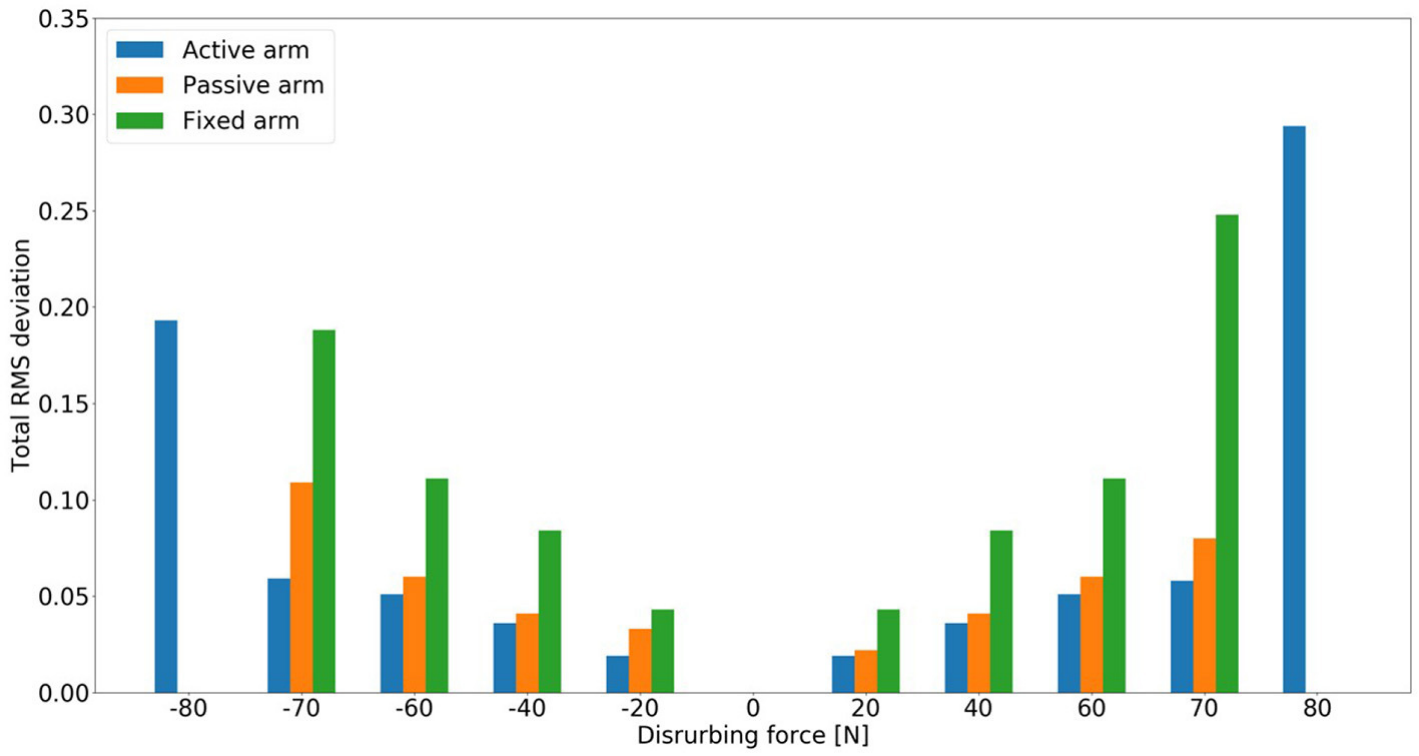
Evolution of the total RMS deviation of the model for three different arm states (active arm, passive arm, and fixed arm) for different disturbing forces: (1) Push backward: −20 [*N*], −40 [*N*], −60 [*N*], −70 [*N*], and −80 [*N*]; (2) No force: 0 [*N*]; (3) Push forward: 20 [*N*], 40 [*N*], 60 [*N*], 70 [*N*], and 80 [*N*]. There was no solution for the cases of passive and fixed arms under the disturbing forces −80 [*N*] and 80 [*N*].

The evolution of the total RMS deviation of the model for the three different arm states (active arm, passive arm, and fixed arm) for different disturbing forces is illustrated in Figure 7. Here, the total RMS deviation is defined to represent the body motion intensity. Figure 7 shows that the total RMS deviation of the balance recovery motion with active arms is less than that with passive arms. Furthermore, the total RMS deviation of the balance recovery motion with passive arms is less than that with fixed arms for the following disturbing forces: −20 [*N*], −40 [*N*], −60 [*N*], −70 [*N*], 20 [*N*], 40 [*N*], 60 [*N*], and 70 [*N*]. This indicates that arm movements contribute to human body balance control and reduce the motion intensity of the hip joint. This conclusion in accordance with the one obtained from a human experiment (Boström et al., 2018). Besides, it is worth to note that the proposed model based on NMPC can recover after a wide range of perturbations; therefore, the robustness of the NMPC is verified as well. This is one of the advantages of the proposed controller with active arm usage.

**Figure 8:**
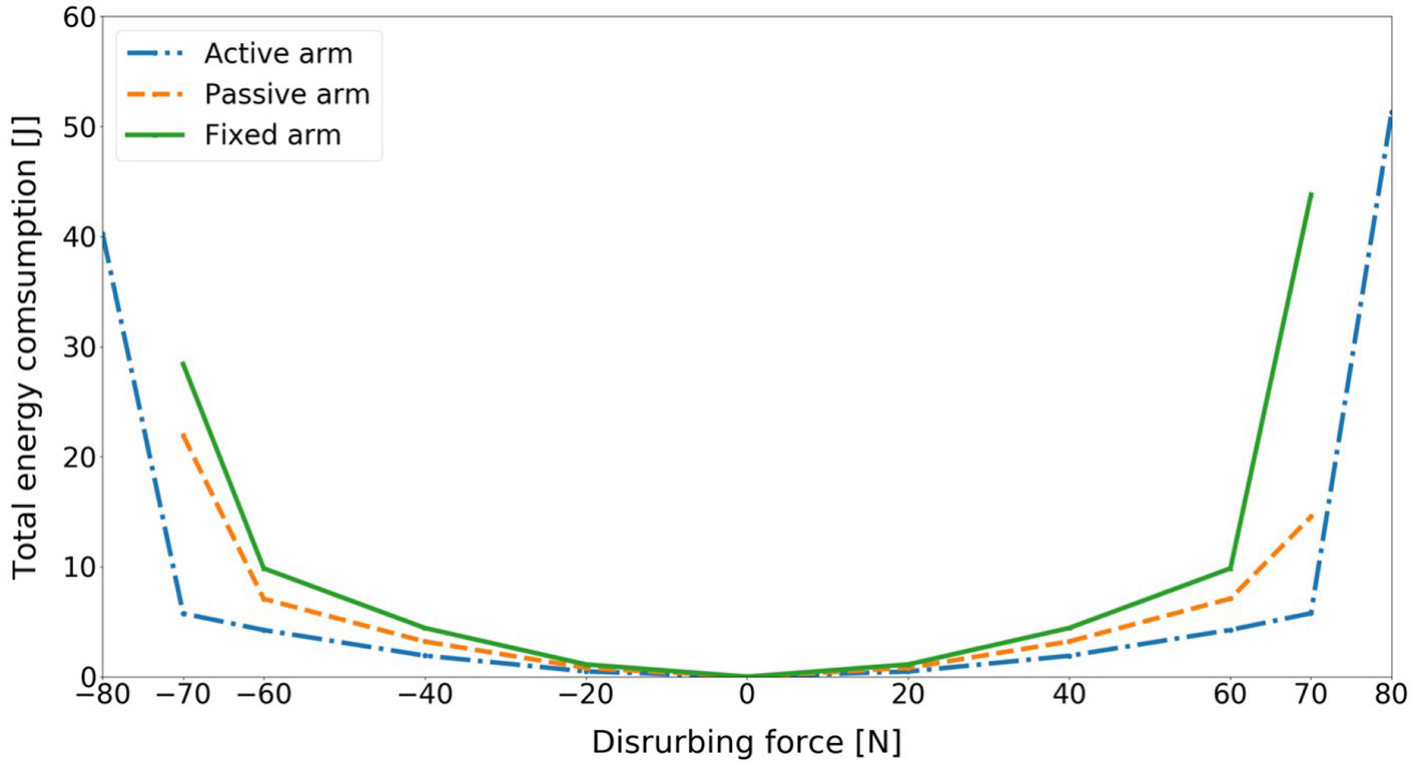
Comparison of total energy consumption of the model for three different arm states (active arm, passive arm, fixed arm) for different disturbing forces: (1) Push backward: −20 [*N*], −40 [*N*], −60 [*N*], −70 [*N*], and −80 [*N*]; (2) No force: 0 [*N*]; (3) Push forward: 20 [*N*], 40 [*N*], 60 [*N*], 70 [*N*], and 80 [*N*].

Figure 8 compares the energy consumption of the model for three different arm states and for different disturbing forces. The energy consumption in this research is joint mechanical energy, which can be computed through the total joint actuator energy consumption of ankle, hip, arms. First, we observe that as the disturbing force intensifies, the balance recovery motion consumes more energy for each case. Most importantly, for the same amount of push, the energy consumption for the balance recovery of the model with active arm rotation is the least followed by passive arm rotation. It is the biggest in the case without arm rotation. This indicates clearly that balance recovery with arm strategy can reduce energy consumption, which is human-like energy-efficient. Humans also optimize the motion behavior for balance recovery to save energy. Thus, the contribution of arm usage to human balance recovery can also be acknowledged from the perspective of energy-efficiency.

**Figure 9:**
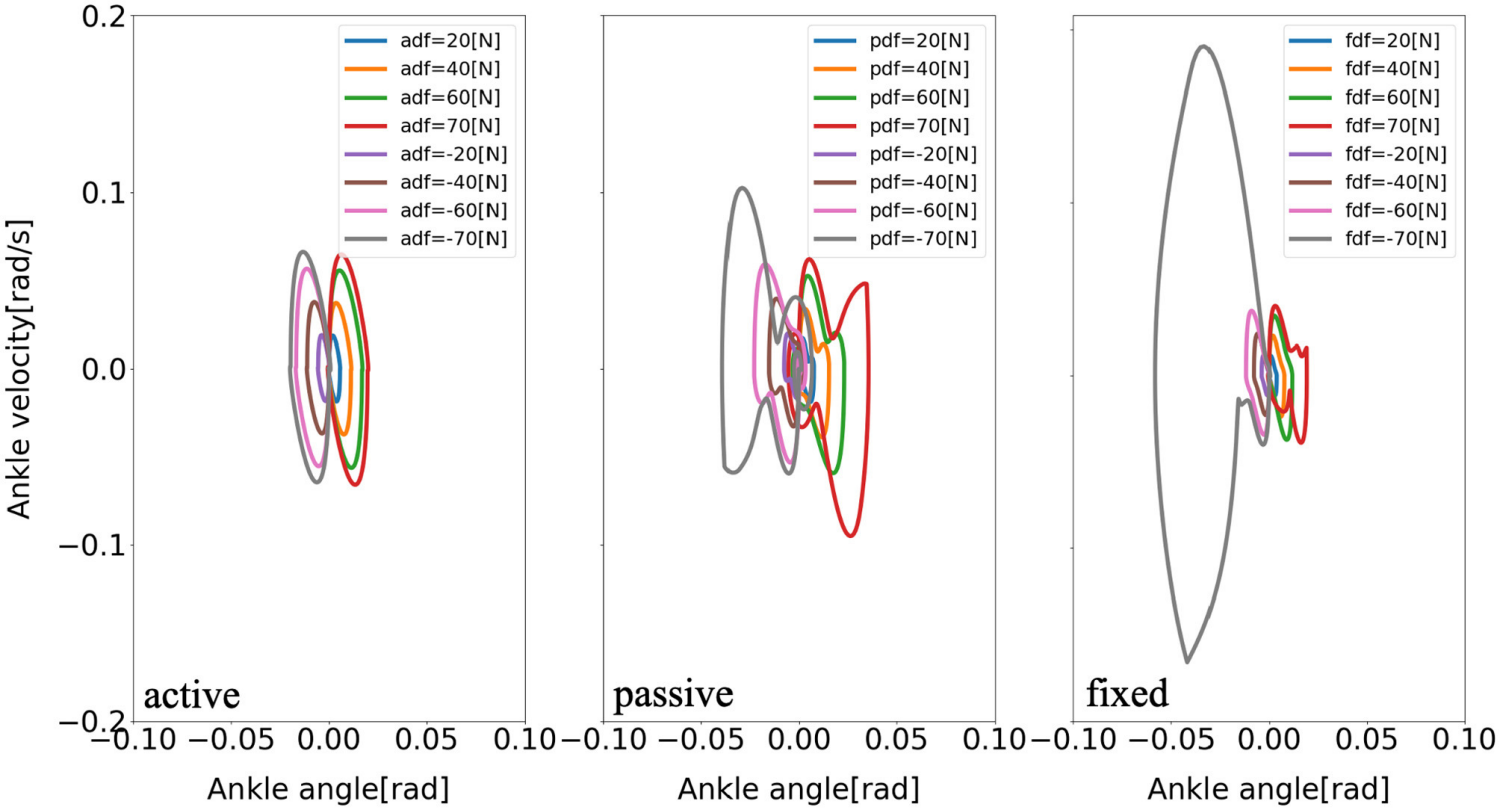
Evolution of the ankle phase portrait of the model for three different arm states (active arm, passive arm, fixed arm) for different disturbing forces: (1) Push backward: −20 [*N*], −40 [*N*], −60 [*N*], and −70 [*N*]; (2) No force: 0 [*N*]; (3) Push forward: 20 [*N*], 40 [*N*], 60 [*N*], and 70 [*N*]. “a,” “p,” and “f” in the labels “adf,” “pdf,” and “fdf” represent the cases with active arms, passive arms, fixed arms, respectively, and “df” represents the disturbing forces.

**Figure 10:**
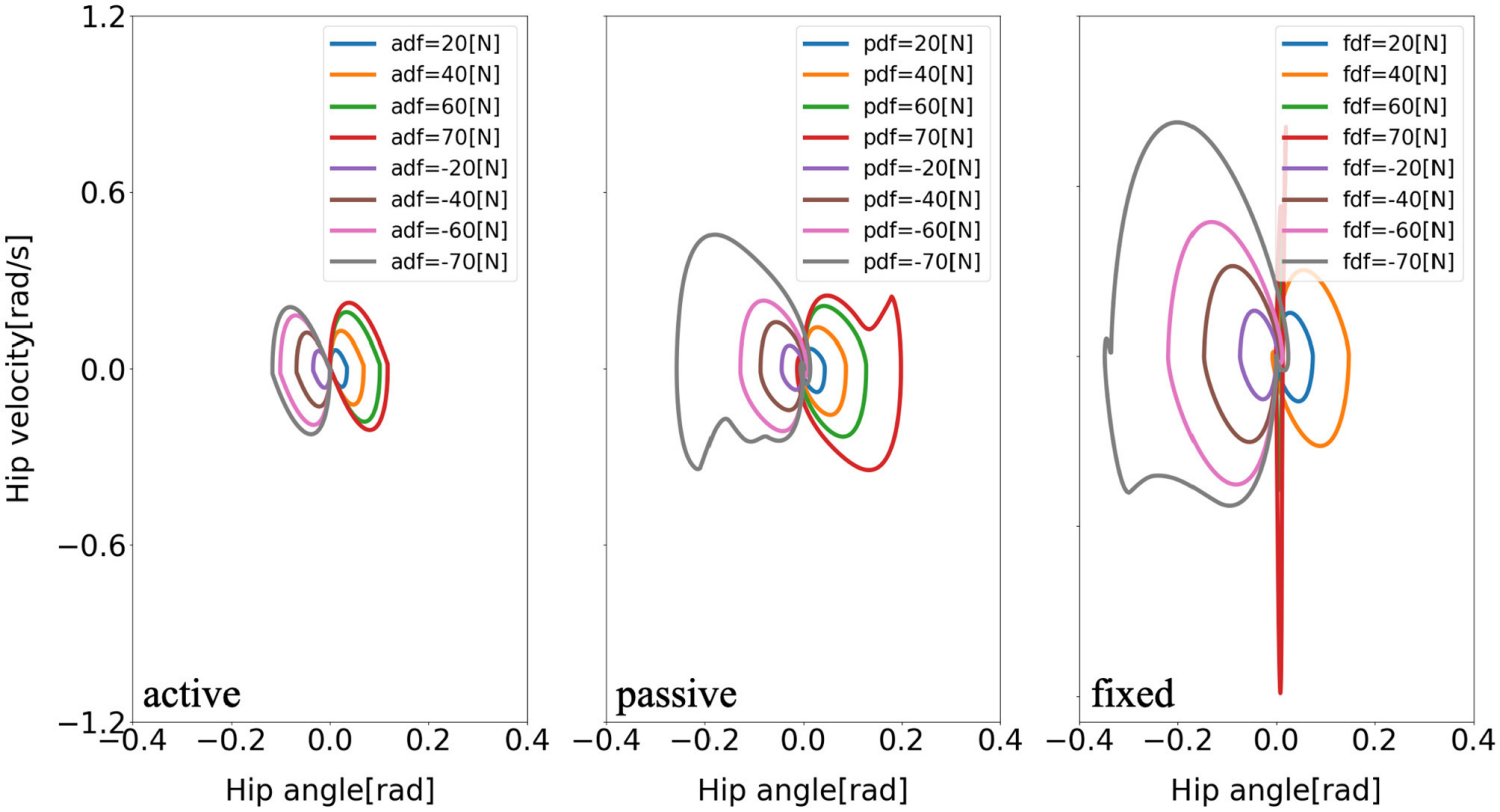
Evolution of the hip phase portrait of the model for three different arm states (active arm, passive arm, fixed arm) for different disturbing forces: (1) Push backward: −20 [*N*], −40 [*N*], −60 [*N*], and −70 [*N*]. (2) No force: 0 [*N*]. (3) Push forward: 20 [*N*], 40 [*N*], 60 [*N*], and 70 [*N*]. “a,” “p,” and “f” in the labels “adf,” “pdf,” and “fdf” represent the cases with active arms, passive arms, fixed arms, respectively, and “df” represents the disturbing forces.

**Figure 11:**
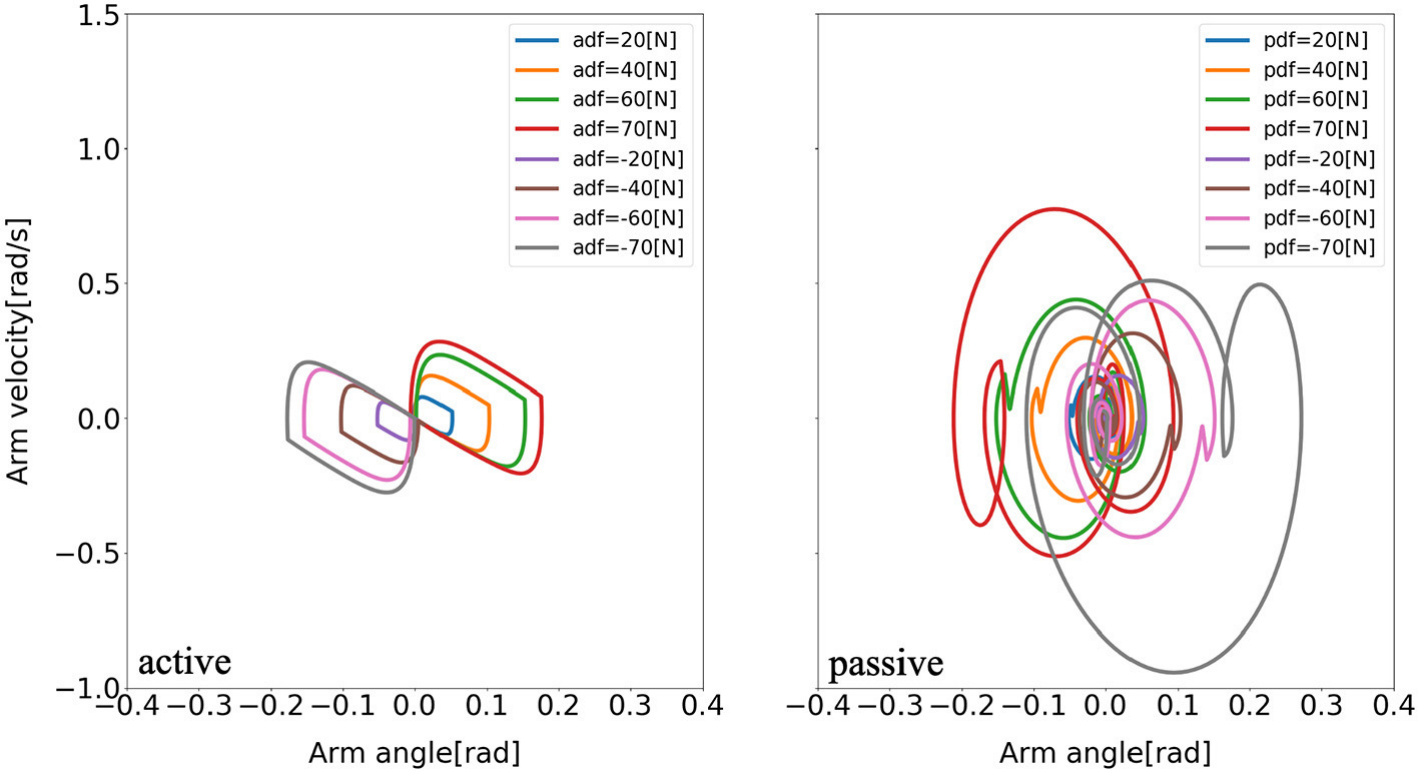
Evolution of the arm phase portrait of the model for two different arm states (active arm, passive arm) for different disturbing forces: (1) Push backward: −20 [*N*], −40 [*N*], −60 [*N*], and −70 [*N*]. (2) No force: 0 [*N*]. (3) Push forward: 20 [*N*], 40 [*N*], 60 [*N*], and 70 [*N*]. “a” and “p” in the labels “adf” and “pdf” represent the cases with active arms and passive arms, respectively, and “df” represents the disturbing forces.

Furthermore, there are consistent limit cycles of the balance recovery for the model with active arm usage over the different disturbing forces, indicating natural temporal regulation on the coordination of ankle, hip, and arm joints, respectively, in Figures 9–11. It means that there is temporal pattern to make compensation against disturbing forces, which can be viewed as there is control strategy since it forms similar form of phase portrait. It implies there is consistent ankle-hip-arm control strategy for active arm usage. However, it is noticeable that portrait form is largely deformed for the passive and fixed arm cases.

**Figure 12:**
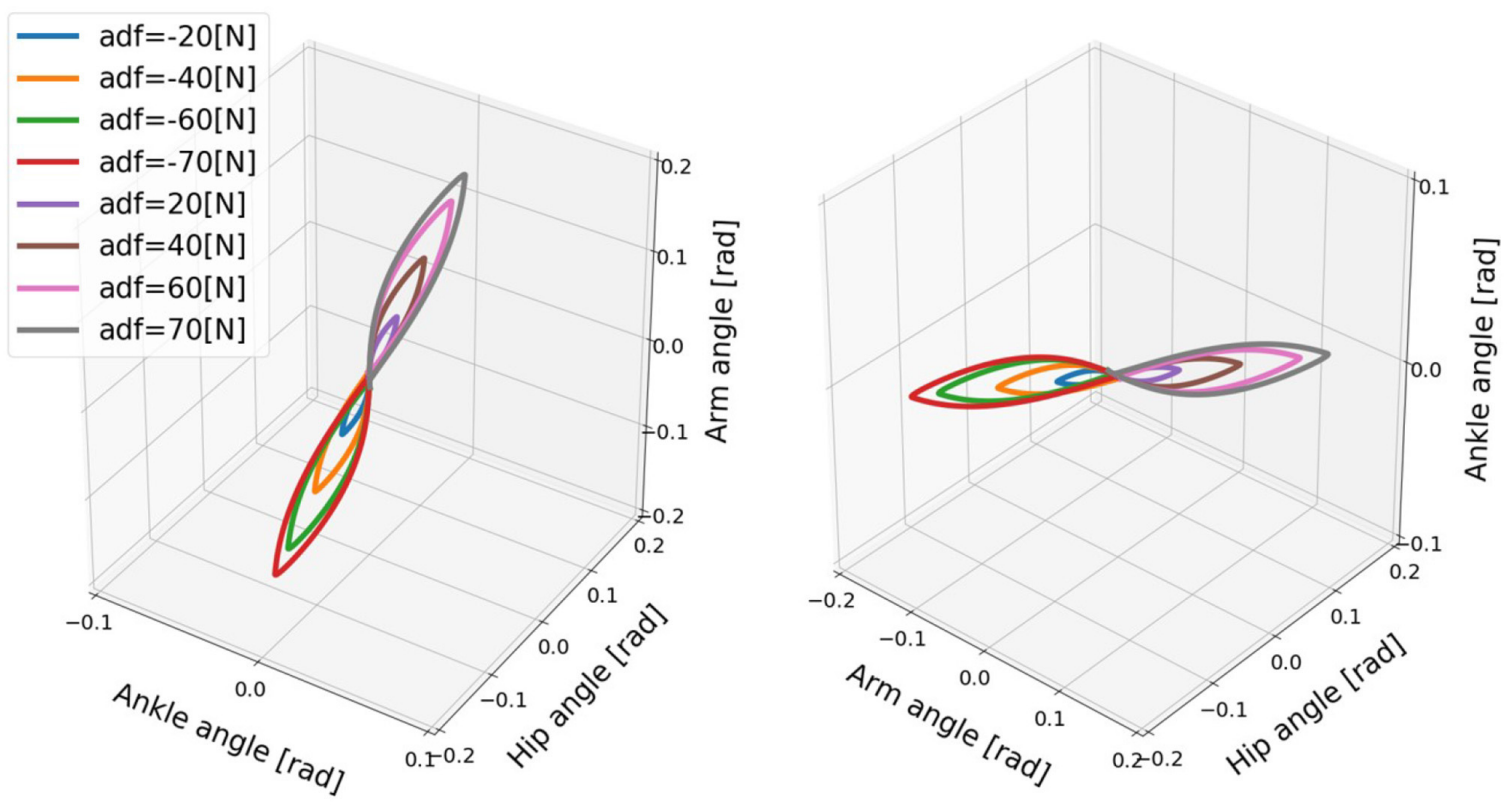
Evolution of the ankle, hip, and arm angles of the model for active arms for different disturbing forces: (1) Push backward: −20 [*N*], −40 [*N*], −60 [*N*], −70 [*N*]. (2) No force: 0 [*N*]. (3) Push forward: 20 [*N*], 40 [*N*], 60 [*N*], 70 [*N*]. “a” in the label “adf” represents the case with active arms, and “df” represents the disturbing force. The mean joint correlation between ankle and hip is 0.898 and the one between hip and arm is 0.966.

Similarly, the relationship of the ankle, hip, arm angles shows aligned spatial pattern over the joints, which represents synergetic joint coordination, and the maximum deviations exhibit linear approximations in Figure 12. The joint correlation of neighboring joints, such as ankle and hip, hip and arm, under the different disturbing forces are computed for synergy existence confirmation (Latash and Zatsiorsky, 2016). The mean joint correlation between ankle and hip is 0.898 and the one between hip and arm is 0.966. Thus, the balance motion with active arm rotation is highly coordinated, which means there is a good synergy performance. The synergy pattern here can represent the ability of task sharing and balance stabilization. However, the motions of balance recovery for the cases of the model with passive and fixed arms did not exhibit similar synergy performance because of the absence of certain patterns. From the synergy analysis perspective, the balance recovery for the model with active arm usage is better than that for the other two cases.

Table 3 shows the contribution of active arm usage to the balance recovery under different ankle capacities. Since ankle is most common injured body site (Fong et al., 2007), we want to observe how arm usages improve the ability of balance maintenance. Here, five cases are considered through different ankle torque constraints (tc) and disturbing forces (df) (Negahban et al., 2013):

1. tc = [−80, 80] [*Nm*], df = 56 [*N*],
2. tc = [−100, 100] [*Nm*], df = 56 [*N*],
3. tc = [−120, 120] [*Nm*], df = 56 [*N*],
4. tc = [−100, 100] [*Nm*], df = 70 [*N*],
5. tc = [−120, 120] [*Nm*], df = 70 [*N*].

**Table 3:** Contributions of active arm usage to balance recovery under different ankle capacities.

**Case**	**Recovery time [s]**	**Arm RMS deviation**	**AEC [J]**	**TEC [J]**
(1)	5.53	0.505	31.681	74.284
(2)	3.2	0.071	0.81	3.11
(3)	3.2	0.071	0.796	3.097
(4)	5.31	0.523	29.914	85.602
(5)	3.32	0.087	1.248	4.824

*Five cases are defined as follows: (1) tc = [−80, 80] [Nm], df = 56 [N]; (2) tc = [−100, 100] [Nm], df = 56 [N]; (3) tc = [−120, 120] [Nm], df = 56 [N]; (4) tc = [−100, 100] [Nm], df = 70 [N]; (5) tc = [−120, 120] [Nm], df = 70 [N]. AEC represents arm energy consumption, and TEC represents total energy consumption*.

Comparing the above ankle boundary constraint settings, we can note that the ankle capacity of case (1) is weaker than that of cases (2) and (3). For the same disturbing force df = 56 [*N*] applied on the center of mass of the upper body, in cases (1), (2), (3), the ankle capacity of case (1) reaches the maximum limit. Therefore, this model needs more efforts for balance recovery. Besides, a longer recovery time and a higher energy consumption are required for case (1), compared with those for cases (2) and (3). For a limited ankle capacity, such as in case (1), the active arm RMS deviation is seven times those in cases (2) and (3), and arm energy consumption is approximately 39 times that in cases (2) and (3). Similarly, for a same disturbing force of 70 [*N*], the active arm RMS deviation in case (4) is six times that in cases (2) and (3), and the arm energy consumption is approximately 24 times that in cases (2) and (3). These observations show that for a limited ankle capacity, arm rotation makes more effort for balance recovery. Furthermore, the ankle capacity in cases (2) and (3) for a disturbing force df = 56 [*N*] does not reach the maximum limit, and the movements of balance recovery are almost the same. For the same ankle capacity in cases (2) and (4), the disturbing force df = 70 [*N*] in case (4) makes the ankle capacity reaching the maximum limit, and active arms need more effort for balance recovery than that in case (2). By comparing cases (3) and (5), although the ankle capacity does not reach the maximum limit in both cases, more efforts are required for a bigger disturbing force.

In the simulation study, various indexes are verified to evaluate the capability of balance recovery. The obtained data indicate that balance recovery with active arms is the most effective strategy, and balance control with arm usage is better than that without arm usage. Besides, Phase portraits of joint angles are considered to analyze the control pattern of balance recovery motion. Furthermore, Ankle torque boundary constraints are set with different values. The relationship between ankle capacity and active arm usage is discussed since in our daily life ankle is easy to be injured, we want to observe how arm usages contribute to balance in this case. Regarding the comparison of our work with previous studies dealing mainly with human balance control without arm strategy, it is worth to point out that in our study we considered three cases including (i) active, (ii) passive, and (iii) fixed arms. This last one corresponds to the case without arm strategy from the literature. Indeed, the obtained results show clearly that the balance model with active arm strategy leads to a less energy consumption, a more robust control, a more synergetic motion, and an improved balance ability, compared to the case without arm strategy.

### 3.3. Human Experimental Setting

Now, we apply three different magnitudes of a pushing force, namely, small push, medium push, and large push, to the backs of the subjects to observe the contributions of arm rotation to human quiet standing balance recovery. The magnitudes of the pushing forces are distinguished by the maximum position deviation of the marker on the subject's neck, and ground reaction force measured by two AMTI force plates. Furthermore, it is important to point out that even though the same pushing force is applied to all the subjects, there is no guarantee that the balance behavior of the subjects would be exactly the same. Consequently, we decided to quantify the levels of this pushing force and classify them into three levels (small, medium, and large). The key point behind this is to distinguish the subjects' balance recovery behavior based on these different levels of the pushing force. The subjects were five healthy men [mean age (25 ± 5) years, mean height (175 ± 10) cm, mean weight (70 ± 10) kg] without any known motor or neurological impairment. The protocols of human experiments were designed according to the Declaration of Helsinki and approved by the Tohoku University ethics committee. In fact, the human experiments have been conducted in two main stages. During the first one, dealing with a pre-training, the subjects are pushed with different forces (according to the three levels explained above, respecting the order : small, then medium, then large for security purposes) to learn how to maintain their balance. During the second stage, dealing with the final experimental tests, the previous different pushing force levels are considered, while disturbing the subjects in stand-up positions, and their behavior data are recorded. The exact spot of the push force is the upper back of the subject. For each level push force, five repetitions are performed for a single subject. The motion of the subject is tracked using 42 markers in the Optitrack system with eight cameras and the ground reaction forces are measured using two force-plates. Then, we export the tracking data of the motion and ground reaction forces and convert them to a standard data format, which can be used in OpenSim (Rajagopal et al., 2016). Then, we obtain the joint angles and torques for each subject through model scaling, inverse kinematics, and dynamics in OpenSim. Here, the inverse dynamics could be solved by using the top-down method. These results can be used to analyze the balance recovery motion and the functions of the ankle, hip, and arm for different magnitudes of disturbing forces.

### 3.4. Comparison With Human Experimental Results

**Figure 13:**
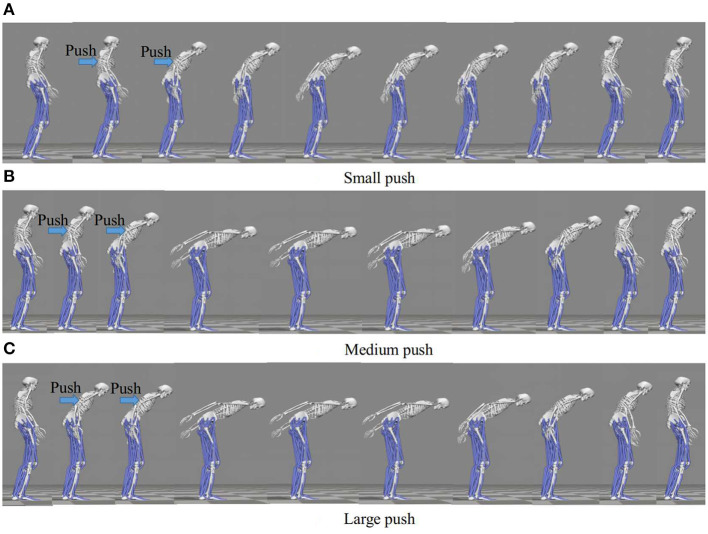
Balance recovery motion of Subject 1 for three different magnitudes of push: (*a*) Small push, (*b*) Medium push, and (*c*) Large push. As the push force increases, more arm usage can be recruited to improve the balance maintenance ability. And, anti-phase exits between arm and hip joint angles, indicating the active arm rotations for balance control motion.

**Figure 14:**
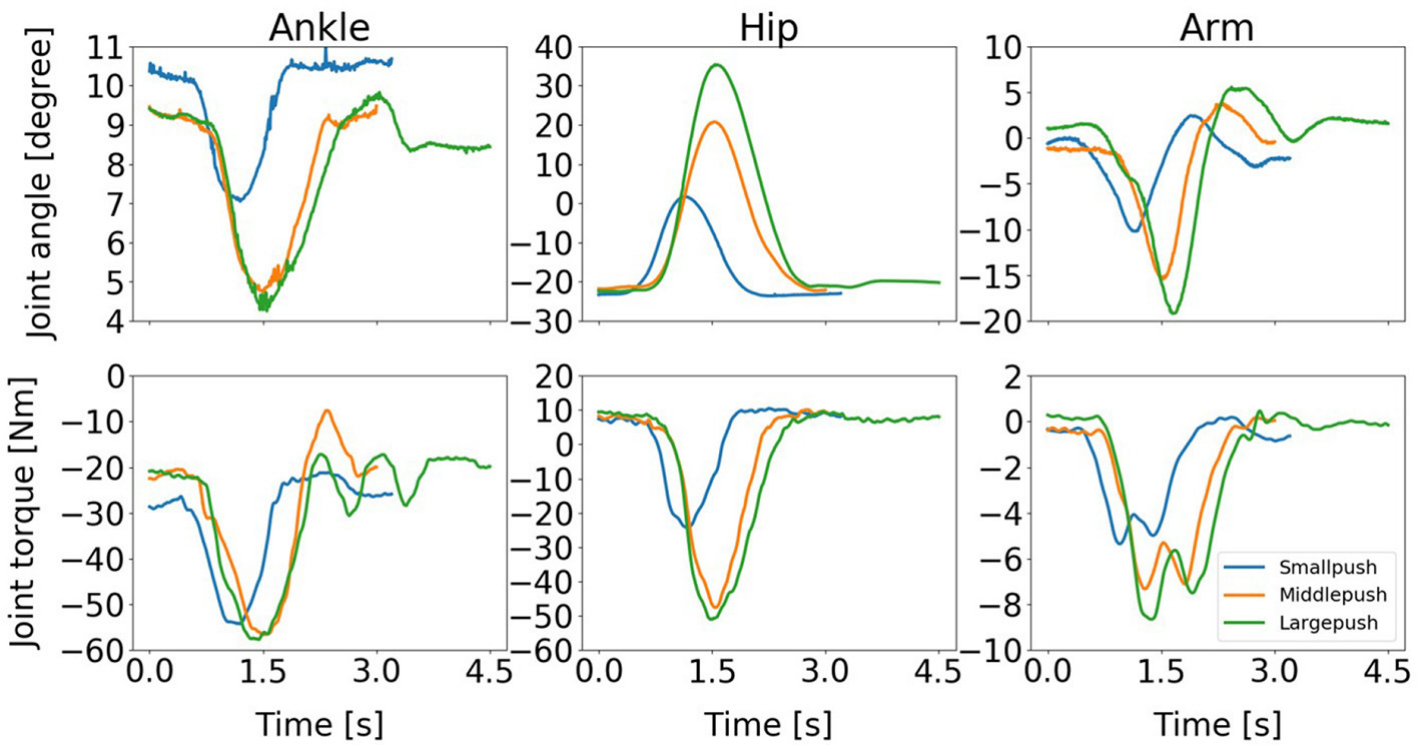
Evolution of the joint angles and torques of the ankle, hip, and arm for three different magnitudes of push: (1) Small push, (2) Medium push, and (3) Large push. It is important to note that ankle joint angle and torque don't change from middle push to large push. It implies necessity of arm usage after ankle usage saturation.

Our discussion in this paragraph focuses on the representative movements on the Subject 1 since the trends discussed for this subject are consistent across all the subjects. The balance recovery motion of Subject 1 for three different magnitudes of push: (a) Small push, (b) Medium push, and (c) Large push is shown in Figure 13. Here, the active arm usage of subject 1 is in a good accordance with the one obtained in our simulation shown in Figure 4 reproduced by the proposed NMPC, where anti-phase between arm and hip joint angles. Besides, as the push force increases, more arm usage can be recruited to improve the balance maintenance ability. Figure 14 shows the evolution of the joint angles and torques of the ankle, hip, and arm for the three different magnitudes of push. The ankle joint angles change slightly, which is similar with the simulation results illustrated in Figure 9, because of the structural limitation of the ankle joint compared to other joints. It is important to note that ankle joint angle and torque don't change from middle push to large push. It means ankle usage meets saturation due to its limited capacity. We have observed this phenomenon also in the simulation study. The hip joint rotates by a larger degree as the magnitude of the pushing force increases. This illustrates that hip joints play a major role in balance recovery. Furthermore, the deviation of the arm joint angles and torques increases. This is because when the magnitude of the pushing force increases, subject 1 attempts to recover balance through more efforts of the arm rotation. From Figure 14, we note that subject 1 spends a longer time in recovering balance for the large push. Table 4 presents the mean of the peak-to-peak values of the joint angles and torques of the ankle, hip, and arm of five subjects for different magnitudes of pushing force. Here, the deviation of the arm joint angles and torques is positively correlated with the magnitude of push force. For large push, we can notice that ankle joint angle increases only 1 degree from middle push case. It implies that ankle usage is already near the saturation due to mechanical constraints, thus the arm strategy to compensate disturbance is essential for large push. This process is consistent to the behavior we have observed in the proposed NMPC controller. Consequently, we conclude that active arm usage contributes to balance recovery in human experiment and the consistent behavior between the predictive controller study and human experiment. This indicates arm movements enhance the capability of balance recovery.

**Table 4:** Mean of the peak-to-peak value of the joint angles (Unit: [*degree*]) and torques (Unit: [*Nm*]) of the ankle, hip, and arm for different pushing forces.

**Push magnitude**	**Ankle**		**Hip**		**Arm**	
	**Angle**	**Torque**	**Angle**	**Torque**	**Angle**	**Torque**
Small push	2.35	33.70	14.10	24.03	12.45	4.35
Middle push	4.58	37.82	24.53	47.19	19.54	6.95
Large push	5.51	46.60	44.91	61.80	24.86	8.32

## 4. Conclusions and Future Work

In this study, we built a simplified human model with arms and proposed an NMPC scheme to reproduce human balance behavior with arm usages. Three arm states, active arms, passive arms, and fixed arms, were considered to study the contributions of the arm movements to balance recovery with different magnitudes of a disturbing force during quiet standing. The contribution of arm usage to human balance control was verified by comparing the total RMS deviation of joint angles, and balance control with active arms was found to be the most effective in terms of the energy consumption and the disturbance effect minimization. Furthermore, the synergetic motion pattern was observed with kinematics during balance recovery with active arms while it was confirmed with joint correlation along with the steady smooth limit cycle pattern, and the total energy consumption was compared. Finally, the results of human experiments were compared with simulation to verify that active arm usage contributes to balance recovery. Our future work may focus on conducting more human balance recovery experiments and analyzing the synergy of body motion at the kinematic, kinetic, and muscle levels. This will help us to gain a better understanding of the mechanism of quiet standing balance with arm strategy and to develop an effective balance controller for rehabilitation.

## Data Availability Statement

The original contributions generated for the study are included in the article/supplementary material, further inquiries can be directed to the corresponding author/s.

## Ethics Statement

The studies involving human participants were reviewed and approved by Tohoku University ethics committee. The patients/participants provided their written informed consent to participate in this study.

## Author Contributions

KS, AC, and MH designed the study. KS implemented the experiments and processed the data. KS wrote the manuscript with the support of AC and MH. All authors have made contributions to the study and approved it for publication.

## Conflict of Interest

The authors declare that the research was conducted in the absence of any commercial or financial relationships that could be construed as a potential conflict of interest.
